# The association between violence exposure and general and cause-specific mortality in people using mental health services: cohort study

**DOI:** 10.1192/bjo.2025.10938

**Published:** 2026-01-12

**Authors:** Nabihah Rafi, Robert Stewart, Amelia Jewell, Hitesh Shetty, Vishal Bhavsar

**Affiliations:** King’s Women’s Mental Health, The David Goldberg Centre for Health Services and Population Research, Institute of Psychiatry, Psychology and Neuroscience, King’s College London, London, UK; South London and Maudsley NHS Foundation Trust, London, UK

**Keywords:** Mortality, mental illness, violence, health inequalities

## Abstract

**Background:**

Many studies have observed a link between mortality and mental illness, although the contribution of violence exposure to mortality in people with mental illness remains under-researched.

**Aims:**

To examine the association of violence exposure, such as being physically assaulted, with general and cause-specific mortality in a population using mental health services.

**Method:**

We assembled a cohort study using electronic health records from a mental health and substance use treatment provider in south-east London. Records were linked to acute medical admission and emergency department presentation data, as well as to a national mortality register with death certificates for deaths registered in England and Wales. Cox regressions estimated the associations of binary and cumulative violence exposure, as indicated by assault admission and presentation to emergency departments for violence-related reasons. Mortality was adjusted for sociodemographic and clinical potential confounders.

**Results:**

The hazard ratio for assault admission with all-cause mortality was 2.14 (95% CI: 1.93–2.36) following covariate adjustment. Adjusted associations were also found with mortality from the following causes: internal (natural) (hazard ratio 1.72, 95% CI: 1.50–1.98), external (hazard ratio 1.94, 95% CI: 1.51–2.48), suicide (hazard ratio 2.20, 95% CI: 1.38–3.52), respiratory (hazard ratio 2.01, 95% CI: 1.41–2.85), circulatory (hazard ratio 1.71, 95% CI: 1.27–2.28), diabetes-related (hazard ratio 2.86, 95% CI: 1.20–6.86) and alcohol-related (hazard ratio 1.56, 95% CI: 1.10–2.22). Results for cumulative assault were consistent with these in both direction and magnitude. There was evidence for an association of weapon-related assault admission with all-cause mortality (hazard ratio 1.58, 95% CI: 1.14–2.18).

**Conclusions:**

People with mental illness, who are exposed to assault, experience greater mortality than those who are not exposed. Excess mortality attributable to violence exposure in people with mental illness was related to deaths from natural and external causes.

Increased mortality in people with mental illness compared with the general population is well established in previous research,^1,2^ as well as in more recent studies.^3^ Increased mortality in mental illness remains evident in recent data from studies investigating COVID-19-associated mortality in individuals with mental illness compared with the general population.^4^ Evidence is limited on effective interventions to reduce excess mortality in this population.^5^ People with mental illness die more commonly by suicide and homicide compared with others,^6,7^ but also die prematurely from many natural causes.^8^ Precise explanations for this inequality remain unknown but, as candidate explanations, investigators have advanced greater unhealthy lifestyles, including smoking^9,10^ and genetic/familial confounding factors.^11^ People with mental illness are also exposed to greater individual and neighbourhood socioeconomic disadvantage, which is strongly associated with mortality.^12,13^

## Exposure to violence

Violence, defined as the intentional use of physical force or power that results in, or has a high likelihood of resulting in, injury, death, psychological harm, maldevelopment or deprivation,^14^ is an important determinant of physical and mental health/illness.^15^ This may be threatened or actual force or power, against oneself or a group or community. Violence is a cause of significant burden and cost to health systems and wider society.^16^ Incidents of exposure to violence through physical or sexual violence, the majority of which do not lead directly to mortality, are still associated with mortality in general population studies.^17,18^ Experience of violence is more prevalent in people with mental illness compared with the general population.^19,20^ Younger age, Black and minority ethnic group, single marital status and socioeconomic deprivation are important risk factors for violence exposure in people with mental illness.^21^ Research has indicated increased levels of premature mortality in individuals who have experienced violent victimisation compared with those who have not.^22^ This was also observed in a mental health population in Sweden,^17^ although research remains limited. The involvement of weapons in violent incidents increases morbidity and mortality,^23^ and there may be a distinct impact on health of violence involving weapons, compared with other types of violence.^24^

## Mortality and exposure to violence

Evidence for a relationship between violence exposure and mortality in people with mental illness could be important in regard to developing and targeting new interventions to improve health outcomes in this group. While an association between violence exposure and non-suicide external mortality is consistent with previous research,^22^ a similar association with mortality from natural causes has been previously documented but has not been tested in people diagnosed with mental illness.^25,26^ Violence exposure might lead to greater mortality due to natural causes, via the accumulation of greater stress and consequent disruption of the hypothalamic–pituitary–adrenal axis^27^ or through greater risky behaviour (smoking and alcohol use) as a result of violence exposure.^28,29^ Thus, we aimed to investigate the association between violence exposure and mortality in people diagnosed with mental illness.

## Method

### Study design

We assembled a cohort study using data extracted from a large electronic mental health record data resource, following up all patients in receipt of mental health care from 1 January 2015 until either date of death or end of study, on 14 November 2022. Experience of violence exposure was evaluated during mental health service receipt as the exposure of interest, for the occurrence of all-cause and cause-specific mortality.

### Data sources and setting

We extracted deidentified clinical data on patients using National Health Service (NHS) mental health services at the South London and Maudsley NHS Foundation Trust (SLaM), which provides comprehensive mental health services to a geographic catchment of over 1.2 million residents in four south London boroughs – Croydon, Lambeth, Lewisham and Southwark. Data on SLaM patients generated through routine clinical care delivered since 1 January 2006 are made available in deidentified form for approved research projects via the SLaM Clinical Record Interactive Search (SLaM-CRIS) platform.^30^ Linked data from a range of other databases are available on patient records in SLaM-CRIS, including those on acute medical hospital care use, and on mortality, as used by this study and further described below.

### Study population

We defined a cohort of patients in SLaM-CRIS with any mental health diagnosis (ICD-10 codes F00–99) assigned at any time from 1 January 2007 onwards. All alive patients in SLaM-CRIS on 1 January 2015 were entered into the observed cohort.

### Measurements

#### Exposure

Violence exposure was measured in the study population by gathering information on receipt of acute medical hospital and emergency department care for violence, using linkage of the SLaM-CRIS database with both the Hospital Episode Statistics admitted patient care data-set (HES-APC) and the accident and emergency data-set (HES-AE).^31^ Both were provided by NHS England, and contain records of all NHS hospitalisations in England. Further information regarding diagnostic codes used to define the exposures are shown in the supplementary material available at http://doi.org/10.1192/bjo.2025.10938.

Four exposure variables were derived: (a) a binary indicator for hospital admission for assault; (b) a score variable for number of hospital admissions for assault; (c) a binary indicator for emergency department presentation for a violence-related reason (described in ref. ^32^); and (d) a score variable for the number of emergency department presentations for a violence-related reason. In addition, we derived a variable for exposure to weapon-related assault based on diagnostic codes, in line with previous literature.^33^

#### Outcome

Both date and cause of death were extracted from linked death certification data obtained from the Office for National Statistics.^34^ Primary causes of death were classified into a broad, upper-level categorisation of natural and external deaths, and further classified into a lower-level categorisation consisting of suicide, non-suicide external, COVID-19-related, neoplastic, respiratory, cardiovascular, diabetes-related and alcohol-related mortality. The diagnostic codes used to define outcomes are listed in the supplementary material.

#### Covariates

Measurements of gender, ethnicity (grouped into White, Black, Asian, mixed and other) and marital status (married/cohabiting, single, divorced/separated and widowed) were taken at the point of first referral to SLaM from structured electronic health record fields. Age was measured at the point of start of cohort observation, which was 1 January 2015 (described further under Analysis). We used structured field data on the first recorded primary psychiatric diagnosis, classified according to ICD-10 chapter (F00–09, F10–19, F20–F29, F30–39, F40–49, F50–59, F60–69, F70–79, F80–89 and F90–98). Substance use comorbidity was measured using a binary variable for the receipt of a primary or secondary psychiatric diagnosis of ICD-10 (F10–19) at any time during contact with mental health services. In addition to measurement of the exposures of interest, HES-APC and HES-AE data were also used to define a binary variable for exposure to assault occurring before referral to mental health services (see supplementary material). The presence of violent behaviour risk events, defined as one or more documented incidents of patient behaviour assessed as posing a risk to others, was ascertained using a natural language-processing algorithm based on language rules developed for a previous project.^35^ This algorithm searched for relevant text in descriptions of clinical risk event based on a set of strings (for example, aggress*, assault”, attack*), applied to SLaM-CRIS data from referral onwards; a full list of language rules is included in the supplementary material. Neighbourhood deprivation was measured using the Index of Multiple Deprivation score reported in 2015, and assigned to patients based on their postcode of residence at referral.

### Analysis

Analyses were conducted in Stata version 18 for Windows. Data on patients with observed information on all analysed variables were described (see below for handling of missing data). Patients were entered into a survival analysis for mortality, with entry defined as 1 January 2015 and exit defined as either date of death or the end of the study, which was 14 November 2022. All theorised confounder variables were entered into Cox regression models in a single stage: these were age, gender, ethnicity, marital status, first primary psychiatric diagnosis, substance use comorbidity, violent behaviour, assault admission before referral and neighbourhood deprivation. Neighbourhood deprivation was included as a linear term in regression models (and is reported in deciles in Table 1). For all-cause mortality we applied a Cox regression model, whereas for cause-specific mortality we estimated sub-hazard ratios using competing risk regressions as defined by Fine and Gray,^36^ specifying each cause of death respectively as the outcome of interest and declaring other causes of death as competing risks. To explore the impact of variability in the time period over which exposure to assault was ascertained, as a sensitivity analysis we estimated the association of exposure rate with all-cause mortality in quintiles, based on information on the number of assault admissions and emergency department presentations for a violence-related reason, and the time period elapsed between referral date and start of cohort observation. Results for this analysis estimated the average change from one quintile to the next in the rate of assault admission or emergency department presentation for violence. Periods of time between accepted referral to the study range from 0.002 to 8 years. Counts for exposure assaults ranged from 0 to 14 for assault admission and from 0 to 29 for emergency department presentation. Rates ranged from 0 to 243.52 events per year for assault admission, and from 0 to 365.23 events per year for emergency department presentation for a violence-related reason. We tested violation of the proportional hazards assumption using Schoenfeld residuals, as implemented in Stata’s estat phtest command, finding no violation.

**Table 1:** Descriptive statistics for study population with complete data

Demographics	Overall number in category	Any assault admission for violence		Any presentation to emergency department for violence		Any death	
		*n*	%	*n*	%	*n*	%
Age band at start of observation (years)							
<16	9839	23	0.23	121	1.23	25	0.25
16–24	24 719	489	1.98	2008	8.12	298	0.84
25–34	29 170	756	2.59	2302	7.89	548	1.88
35–44	26 426	682	2.58	1831	6.93	1043	3.95
45–54	23 594	517	2.19	1367	5.79	2073	8.79
55–64	13 433	165	1.23	443	3.30	2193	16.33
65–74	10 002	57	0.57	169	1.69	3408	34.07
75+	16 931	41	0.24	158	0.93	11 103	65.58
Gender							
Women	79 865	704	0.88	2940	3.68	10 575	13.24
Men	74 249	2026	2.73	5459	7.35	10 026	13.50
Ethnicity							
White	100 494	1843	1.83	5460	5.43	15 911	15.83
Mixed	5010	102	2.04	357	7.13	222	4.43
Asian	10 075	128	1.27	425	4.22	1019	10.11
Black	28 625	514	1.80	1705	5.96	2777	9.70
Other	9910	143	1.44	452	4.56	672	6.78
Marital status							
Married/cohabiting	33 550	273	0.81	926	2.76	5845	17.42
Single	99 468	2174	2.19	6658	6.69	7578	7.62
Divorced	12 110	235	1.94	661	5.46	2120	17.51
Widowed	8986	48	1.77	154	1.71	5058	56.29
First primary psychiatric diagnosis (ICD-10)							
F00–09	17 599	118	0.67	323	1.84	7950	45.17
F10–19	25 623	1117	4.36	2643	10.31	2500	9.76
F20–29	11 370	152	1.34	597	5.25	1356	11.93
F30–39	26 353	262	0.99	1086	4.12	2756	10.46
F40–49	22 795	276	1.21	969	4.25	1832	8.04
F50–59	6949	27	0.39	127	1.83	106	1.53
F60–69	2819	92	3.26	260	9.22	191	6.78
F70–79	1725	14	0.81	60	3.48	180	10.43
F80–89	3068	17	0.55	95	3.10	31	1.01
F90–98	35 813	655	1.83	2239	6.25	3699	10.33
Substance use comorbidity							
No	121 186	1238	1.02	4709	3.89	17 325	14.30
Yes	32 928	1492	4.53	3690	11.21	3276	9.95
Violent behaviour risk event							
No	150 670	2616	1.74	8081	5.36	20 260	13.45
Yes	3444	114	3.31	318	9.23	3.41	9.90
Any assault admission before referral							
No	148 737	1785	1.20	3622	2.44	20 149	13.55
Yes	5377	945	17.57	4777	88.84	452	8.41
Decile of neighbourhood deprivation							
1 (most deprived)	8615	221	2.57	671	7.79	1113	12.92
2	39 245	797	2.03	2454	6.25	5201	13.25
3	29 050	607	2.09	1847	6.36	3861	13.29
4	24 747	432	1.75	1327	5.36	3247	13.12
5	17 648	273	1.55	829	4.70	2314	13.11
6	11 110	176	1.58	495	4.46	1537	13.83
7	8061	86	1.07	314	3.90	1030	12.78
8	6162	54	0.88	197	3.20	870	14.12
9	6322	57	0.90	183	2.89	1002	15.85
10 (least deprived)	3154	27	0.86	82	2.60	426	13.51
Column total	154 114	2730	1.77	8399	5.45	20 601	13.37

The original cohort included 289 094 patients however due to missing data on one or more variables, the analysis cohort was reduced to 154 114 patients.

### Missing data

In addition to complete case analyses, we estimated all coefficients of interest using multiple imputation with chained equations generated using the mi command in Stata, in combination with Rubin’s rules.^37^ Analyses was based on 10 imputed data-sets (*n* = 289 094 each). Distributions for imputation were selected based on parameterisation of the missing variable (logistic regression for gender, multinomial logistic regression for ethnicity, marital status and first psychiatric diagnosis, and linear regression for neighbourhood deprivation).^38^

## Results

### Description of cohort

The original data-set included 289 094 patients; however, due to missing data, the analysis cohort included only 154 114. We analysed those patients with complete data on the modelled variables described in Table 1. Men were more likely to be admitted to hospital for assault (2.73%), and to present to an emergency department for a violence-related reason (7.35%), compared with women (0.88 and 3.68%, respectively). Compared with married/cohabiting patients, single individuals experienced the highest prevalence of assault admissions and emergency department presentations for a violence-related reason. Assault admission was most common among those diagnosed with substance use disorders, and least common among those diagnosed with behavioural syndromes associated with physiological disturbances and physical factors, including eating disorders. Presentations to an emergency department for a violence-related reason were more common among those with substance use disorders compared with those without. All-cause mortality was slightly more common in men (13.50%) than in women (13.24%). All-cause mortality was greatest among White patients (15.83%), followed by Asian (10.11%), Black (9.70%), other (6.78%) and mixed (4.43%). Widowed patients experienced the greatest mortality (56.29%), compared with single patients (7.62%). All-cause mortality was highest among those diagnosed with organic mental disorders (45.17%), and lowest among those with disorders of psychological development (1.01%).

### Regression models

All models produced estimates, except for those relating to any weapon-related admission and the number of weapon-related admissions with mortality from a cause not otherwise specified (NOS). For those outcomes, models did not converge owing to small numbers of cases with the outcome. Adjustment for age had a substantial contribution to estimates, with lower attenuation observed following adjustment for other variables. Following adjustments for potential confounders, the following variables were significantly associated with all-cause mortality: admission for assault (hazard ratio 2.14, 95% CI: 1.93–2.36), emergency department presentation for a violence-related reason (hazard ratio 1.58, 95% CI: 1.14–2.18), count variables for number of admissions for assault (hazard ratio 1.40, 95% CI: 1.32–1.50) and number of emergency department presentations for a violence-related reason (hazard ratio 1.20, 95% CI: 1.17–1.24). Adjusted associations were also found with mortality from the following: internal (natural) (hazard ratio 1.72, 95% CI: 1.50–1.98), external (hazard ratio 1.94, 95% CI: 1.51–2.48), suicide (hazard ratio 2.20, 95% CI: 1.38–3.52), respiratory (hazard ratio 2.01, 95% CI: 1.41–2.85), circulatory (hazard ratio 1.71, 95% CI: 1.27–2.28), diabetes-related (hazard ratio 2.86, 95% CI: 1.20–6.86) and alcohol-related (hazard ratio 1.56, 95% CI: 1.10–2.22). Admissions for weapon-related assault were also associated with all-cause mortality (hazard ratio 1.58, 95% CI: 1.14–2.18; see Table 2).

**Table 2:** Cox regression estimates reporting associations of violence exposure with all-cause mortality and mortality from specific causes

Violence exposure	All-cause mortality, hazard ratio (95% CI)	Upper-level categorisation		Lower-level categorisation								
	All causes	Natural, sub-hazard ratio (95% CI)	External, sub-hazard ratio (95% CI)	Suicide, sub-hazard ratio (95% CI)	Non-suicide external, sub-hazard ratio (95% CI)	NOS, sub-hazard ratio (95% CI)	COVID-19, sub-hazard ratio (95% CI)	Cancer, sub-hazard ratio (95% CI)	Respiratory, sub-hazard ratio (95% CI)	Circulatory, sub-hazard ratio (95% CI)	Diabetes-related, sub-hazard ratio (95% CI)	Alcohol-related, sub-hazard ratio (95% CI)
Number of deaths in category	42 293	36 812	3376	1187	2096	813	1292	6602	4846	8902	675	1473
Binary assault admission	2.14 (1.93, 2.36)	1.72 (1.50, 1.98)	1.94 (1.51, 2.48)	2.20 (1.38, 3.52)	1.94 (1.45, 2.59)	2.33 (1.11, 4.87)	1.65 (0.84, 3.26)	1.31 (0.94, 1.84)	2.01 (1.41, 2.85)	1.71 (1.27, 2.28)	2.86 (1.20, 6.86)	1.56 (1.10, 2.22)
Binary ED presentation for violence	1.58 (1.14, 2.18)	1.95 (1.73, 2.20)	2.05 (1.62, 2.58)	1.16 (0.73, 1.83)	2.66 (2.03, 3.47)	4.84 (2.90, 8.07)	1.13 (0.67, 1.89)	1.56 (1.18, 2.06)	1.71 (1.19, 2.46)	1.46 (1.14, 1.88)	1.83 (0.83, 4.02)	2.14 (1.56, 2.94)
Count assault admission	1.40 (1.32, 1.50)	1.37 (1.28, 1.48)	1.33 (1.19, 1.51)	1.4 0 (1.14, 1.72)	1.34 (1.17, 1.55)	1.44 (1.07, 1.95)	1.27 (0.87, 1.85)	1.21 (1.00, 1.46)	1.48 (1.26, 1.72)	1.44 (1.26, 1.66)	1.67 (1.15, 2.43)	1.22 (1.02, 1.45)
Count ED presentation for violence	1.20 (1.17, 1.24)	1.19 (1.16, 1.23)	1.17 (1.10, 1.23)	1.09 (0.93, 1.27)	1.18 (1.12, 1.25)	1.23 (1.14, 1.32)	0.84 (0.61, 1.15)	1.17 (1.07, 1.29)	1.09 (0.99, 1.21)	1.15 (1.08, 1.23)	1.25 (1.11, 1.39)	1.12 (1.05, 1.18)
Weapon-related admission count	1.55 (1.19, 2.02)	1.54 (1.09, 2.17)	1.50 (0.93, 2.40)	1.47 (0.61, 3.56)	1.57 (0.90, 2.75)	–	1.14 (0.22, 6.05)	0.75 (0.27, 2.11)	2.50 (1.51, 4.14)	1.92 (1.08, 3.42)	2.36 (0.66, 8.47)	1.54 (0.65, 3.66)
Weapon-related admission binary	1.58 (1.14, 2.18)	1.51 (1.02, 2.23)	1.62 (0.89, 2.93)	1.73 (0.56, 5.34)	1.67 (0.83, 3.40)	–	1.25 (0.17, 8.92)	0.78 (0.25, 2.42)	3.50 (1.66, 7.37)	1.88 (0.88, 4.01)	3.34 (0.46, 24.56)	1.04 (0.39, 2.77)
Assault admission rate in quintiles	1.09 (1.05, 1.13)	1.06 (1.02, 1.11)	1.18 (1.08, 1.29)	1.22 (1.06, 1.41)	1.18 (1.08, 1.29)	1.16 (0.93, 1.45)	1.00 (0.79, 1.25)	1.01 (0.90, 1.13)	1.12 (1.00, 1.24)	1.10 (1.01, 1.20)	0.98 (0.66, 1.48)	1.14 (1.02, 1.27)
ED presentation rate in quintiles	1.05 (1.02, 1.09)	1.03 (0.99, 1.06)	1.22 (1.14, 1.32)	1.01 (0.87, 1.16)	1.22 (1.14, 1.32)	1.31 (1.12, 1.52)	0.82 (0.67, 1.00)	0.99 (0.91, 1.08)	0.98 (0.89, 1.08)	1.02 (0.94, 1.09)	1.13 (0.89, 1.44)	1.21 (1.12, 1.32)

NOS, not otherwise specified; ED, emergency department.

Estimates based on *n* = 154 114 records with complete data for the modelled variables.

Estimates are adjusted for age (years), gender, ethnicity, marital status, first primary psychiatric diagnosis, substance use comorbidity, violent behaviour risk event, any assault admission prior to referral and neighbourhood deprivation.

Upper-level categorisation of deaths was into natural and external causes; lower-level categorisation of deaths was into suicide, non-suicide external, NOS, COVID-19-related, cancer, respiratory, circulatory, diabetes-related and alcohol-related mortality.

Estimates for assault admission rate and ED presentation rate reflect the increase in hazard ratio (all-cause mortality) and sub-hazard ratios (cause-specific mortality) on moving from one quintile to the next, e.g. from the second to the third.

Admission for assault, emergency department presentation for a violence-related reason and weapon-related admission for assault were all associated with mortality from natural causes. For example, patients experiencing any presentation for a violence-related reason were twofold more likely to experience mortality compared with those not presenting to an emergency department for a violence-related reason. Having an admission for weapon-related assault was accompanied by a 1.51-fold increase in mortality risk over follow-up. Binary and count variables for assault admission and emergency department presentations for a violence-related reason were associated with external causes; however, estimates for weapon-related admission were not statistically significant for this outcome. For suicide mortality, statistically significant associations were found for binary assault admission and the number of assault admissions, but not for emergency department presentation for a violence-related reason, nor weapon-related admission. Any assault admission, and the number of assault admissions, were associated with respiratory mortality, and with any weapon-related assault admission and the number of weapon-related assault admissions. No significant associations were found between any violence exposure variable and COVID-19 mortality. Circulatory mortality was associated with binary and count variables for assault admission, and for presentation to an emergency department for a violence-related reason. An increasing number of weapon-related assault admissions were also associated with circulatory mortality. Binary indicators for assault admission, and for emergency department presentation for a violence-related reason, were associated with diabetes-related mortality. Binary and count indicators for assault admission were associated with alcohol-related mortality. Binary and count indicators for emergency department presentation for a violence-related reason were also associated with alcohol-related mortality. Sensitivity analysis, testing the association of exposure rate in quintiles with mortality, found associations with the same direction as the main analyses (see Table 2). Association estimates from complete case analysis (*n* = 154 114) generally agreed in direction and magnitude with results from analyses of multiple imputed data (based on 10 imputed data-sets, *n* = 289 094 each) (see Table 3).

**Table 3:** Multiple imputation-based estimates from Cox regressions reporting associations of violence exposure with mortality

Violence exposure	All-cause mortality	Upper-level categorisation		Lower-level categorisation								
	All causes, hazard ratio (95% CI)	Natural, hazard ratio (95% CI)	External, hazard ratio (95% CI)	Suicide, hazard ratio (95% CI)	Non-suicide external, hazard ratio (95% CI)	NOS, hazard ratio (95% CI)	COVID-19, hazard ratio (95% CI)	Cancer, hazard ratio (95% CI)	Respiratory, hazard ratio (95% CI)	Circulatory, hazard ratio (95% CI)	Diabetes-related, hazard ratio (95% CI)	Alcohol-related, hazard ratio (95% CI)
Binary assault admission	1.91 (1.73, 2.11)	1.76 (1.56, 1.99)	2.26 (1.85, 2.75)	2.05 (1.39, 3.02)	2.44 (1.93, 3.08)	2.54 (1.40, 4.61)	1.36 (0.73, 2.53)	1.19 (0.88, 1.62)	2.20 (1.62, 2.98)	1.75 (1.35, 2.26)	2.89 (1.33, 6.28)	1.69 (1.26, 22.7)
Binary ED presentation for violence	2.26 (2.09, 2.43)	2.24 (2.02, 2.50)	2.50 (2.07, 3.01)	1.37 (0.92, 2.05)	3.21 (2.59, 3.99)	4.56 (2.85, 7.30)	1.37 (0.78, 2.41)	1.80 (1.42, 2.29)	2.32 (1.74, 3.10)	1.72 (1.35, 2.20)	2.25 (1.08, 4.68)	2.22 (0.59, 1.15)
Count assault admission	1.32 (1.27, 1.37)	1.30 (1.23, 1.37)	1.35 (1.26, 1.45)	1.37 (1.20, 1.58)	1.35 (1.24, 1.46)	1.38 (1.11, 1.72)	1.12 (0.76, 1.67)	1.13 (0.95, 1.33)	1.37 (1.23, 1.53)	1.33 (1.20, 1.47)	1.45 (1.11, 1.91)	1.22 (1.08, 1.39)
Count ED presentation for violence	1.21 (1.18, 1.24)	1.20 (1.16, 1.24)	1.21 (1.17, 1.26)	1.17 (1.05, 1.31)	1.23 (1.18, 1.28)	1.27 (1.13, 1.44)	0.96 (0.70, 1.33)	1.18 (1.09, 1.27)	1.17 (1.06, 1.29)	1.18 (1.09, 1.27)	1.29 (1.08, 1.54)	1.16 (1.08, 1.24)
Weapon-related admission count	1.66 (1.33, 2.07)	1.51 (1.13, 2.02)	1.85 (1.28, 2.68)	1.38 (0.57, 3.35)	2.10 (1.40, 3.13)	1.29 (0.21, 7.71)	0.88 (0.14, 5.72)	0.59 (0.20, 1.78)	2.29 (1.27, 4.14)	1.86 (1.11, 3.10)	2.13 (0.40, 11.18)	1.50 (0.81, 2.78)
Weapon-related admission binary	1.71 (1.30, 2.25)	1.51 (1.06, 2.14)	2.00 (1.25, 3.20)	1.60 (0.59, 4.33)	2.30 (1.35, 3.92)	1.44 (0.20, 10.37)	0.92 (0.13, 6.57)	0.60 (0.19, 1.86)	2.94 (1.46, 5.94)	1.85 (0.96, 3.58)	2.71 (0.38, 19.57)	1.10 (0.45, 2.66)

NOS, not otherwise specified; ED, emergency department.

Estimates based on combining estimates across multiply imputed data-sets, each with *n* = 264 049.

Estimates are adjusted for age (years), gender, ethnicity, marital status, first primary psychiatric diagnosis, substance use comorbidity, violent behaviour risk event, any assault admission prior to referral and neighbourhood deprivation.

Upper-level categorisation of deaths was into natural and external causes; lower-level categorisation of deaths was into suicide, non-suicide external NOS, COVID-19-related, cancer, respiratory, circulatory, diabetes-related and alcohol-related mortality.

## Discussion

### Summary of findings

Patients who experienced a hospital admission for assault after their first psychiatric diagnosis had a twofold elevation in mortality risk. This association remained when using emergency department presentations for violence as an alternative measure of violence exposure, and was evident for the number of assault admissions and violence-related emergency department presentations experienced. Violence exposure indicators were associated with both external mortality and mortality from natural causes. Natural cause associations were driven by associations of violence exposure with respiratory, circulatory, diabetes-related and alcohol-related mortality. Weapon-related admissions for assault were also associated with all-cause mortality and natural mortality, but associations with specific causes were inconsistent.

### Interpretation/explanation

The clustering of violence exposure incidents within individuals in the general population is well known,^39^ and is consistent with the relationship between assault hospitalisation and non-suicide external mortality (including homicides and accidental deaths) that we observed in this study. Violence exposure is also a known predictor of death by suicide,^40^ and we reproduced this finding in the current study.

While we observed association between violence exposure and external mortality, we also observed association between violence exposure and mortality from natural causes. For natural causes, our design cannot eliminate the possibility of residual confounding – that is, of association being driven by common causes of both violence exposure and mortality from natural causes, which were not adequately measured in the analysis. People using mental health services and who are exposed to violence may have worse physical health for a range of reasons, such as the metabolic effects of psychiatric treatment, which in turn might influence cause-specific mortality risk (e.g. diabetes-related mortality); we were unable to assess this in our study because we did not have access to information on the distribution of underlying diseases in the exposed and unexposed populations. Violence exposure, including repeated exposure, might influence mortality risk through a reduced capability to manage existing physical illness, increasing the risk of progression of pre-existing morbidity. Violence exposure, including repeated incidents of such exposure, could result in psychological stress that could, over time, cause stress to body systems, as proposed by the allostatic load hypothesis.^27^ Violence exposure could precipitate increase in unhealthy behaviours, including alcohol use and smoking, which are important drivers of mortality in people with mental illness.^28,29^ Violence exposure may be an indicator of relationship stress, which some evidence suggests may affect mortality risk.^41^ Violence exposure, including repeated violence exposure, might influence mortality risk through a reduced capability to manage existing physical illness, increasing the risk of disease progression.

### Previous literature on this topic

Our examination of the association of violence exposure with mortality in people with mental illness extends the existing literature on violence as a mortality risk factor in the general population. Stenbacka and colleagues, in a study of Swedish military conscripts, found a threefold increase in mortality risk in victims of violence compared with non-victims.^18^ In a study of combined Swedish and Finnish national general population registers, Sariaslan et al found that people exposed to violence experienced a 1.9-fold increase in premature mortality compared with those not thus exposed.^42^ Bhavsar et al found association between physical assault victimisation and mortality in Russian men of working age,^25^ and similar associations were reported in an analysis of two British birth cohorts.^43^ The present study highlights excess mortality experienced by people exposed to violence and who are in contact with mental health services.

Violence exposure is an important direct cause of mortality in people with mental illness. For example, Crump and colleagues, in a register-based study in Sweden, found a fivefold increase in death by homicide in people with psychiatric diagnoses compared with the general population.^44^ Hiroeh and colleagues also found an elevated rate of homicide death in people with mental illness in Danish population registers.^7^

Our work extends existing knowledge on the impact of violence on mortality in mental illness, by suggesting that homicide mortality (as characterised in the literature above) may not fully capture the mortality penalty of violence exposure in people with mental illness. This is consistent with evidence that, aside from violent incidents that result in direct mortality, overall violence exposure is more common among people with mental illness than in the general population.^20,45^

### Strengths and limitations

Most estimates were sufficiently precise for statistical inference, and account was taken of major potential confounders. Linked data on hospital use provided comprehensive information on admissions for different causes, and we used two indicators of violence exposure (assault admission and emergency department presentation for violence), for which our model estimates generally agreed.

In common with other healthcare indicators for violence exposure, admission and emergency department presentation data do not capture all violence. This could have led to under-ascertainment of the exposure and, had there been a negative association between violence exposure and mortality, this would have introduced bias into our results. We took account of a range of plausible confounders, including a measure of violence exposure prior to referral into mental health services, in order to account for the clustering of violence exposure incidents over time. This variable is likely to have underestimated the burden of lifetime violence exposure in this population, which experiences a high prevalence of violence, including via physical and sexual violence during childhood.

Our design could not account for exposure to violence after 1 January 2015. We did not have information on the timing of violent risk events in relation to exposure, which may have led to over-adjustment of our estimates if some violent risk events had resulted from violence exposure. Although we adjusted estimates for age in our analyses, we did not evaluate heterogeneity in associations based on age, which exerts a strong influence on mortality.

We ascertained incidents of violence exposure that occurred from the time of referral to mental health services to the start of observation time, which was variable between participants in the sensitivity analysis. This tested the association of rate of assault with that of mortality, suggesting that this was unlikely to have introduced bias. Measurement of suicide based on death certification is liable to a degree of under-ascertainment. Our estimates for COVID-19-related mortality should be treated with caution, given that these are based on an analytic population conditioned on survival until 2020 (the onset of the COVID-19 pandemic).

### Implications

The association we identified between violence exposure and mortality from natural causes in people with mental illness warrants further research attention to understand the contributory pathways, including through shared causes of both violence exposure and mortality. Strategies to improve the identification and assessment of violence exposure in people with mental illness could improve the quality of care by reducing health inequalities, as well as by uncovering wider healthcare needs in patients exposed to violence.

## Supporting information

Rafi et al. supplementary material Rafi et al. supplementary material

## Data Availability

The data that support the findings of this study are available from South London and Maudsley (SLaM) National Health Service (NHS) Foundation Trust, but restrictions apply to the availability of these data, which were used under licence for the current study. Data are not publicly available due to privacy and ethical restrictions. Researchers may apply for access to the data through the SLaM-Clinical Record Interactive Search (CRIS) Oversight Committee, subject to the relevant approvals.

## Supplementary material

The supplementary material is available online at http://doi.org/10.1192/bjo.2025.10938
