# Supplementary material for: The association between violence exposure and general and cause-specific mortality in people using mental health services: cohort study
Source: BJPsych Open. 2026 Jan 12;12(1):e35. doi: 10.1192/bjo.2025.10938 (PMC12835711; doi:10.1192/bjo.2025.10938)
Supplement: Rafi et al. supplementary material [file S2056472425109381sup001.docx]

# SUPPLEMENTary material

Diagnostic codes used to define the exposures

In HES-APC data, we defined violence exposure as admission for ICD-10 diagnostic codes X85 - Y09. This was inclusive of Y06, neglect and abandonment, and Y07 for other maltreatment syndromes, all of which are grouped under the block heading “Assault” in ICD-10. In contrast to the other codes in the block which capture commission of violent acts (e.g. using bodily force or a sharp object), Y06 reflects omission, where harm results from deliberate withholding of care. NHS Digital similarly defines assault-related hospital episodes as admissions with any of these codes X85 -Y09 recorded. Therefore, we included Y06 and Y07 to align with these standard definitions and with previous research using this block of ICD-10 codes, allowing comparison with national statistics and previous research.

To define ED presentations for violence we collected all ED presentations in the HES AE data where the presenting reason was “Violence”.

Diagnostic codes to define outcomes

ICD-10 codes were used to determine the cause of death which were grouped into all-cause (codes A00-R99; U00-Y89), natural cause (A00-Q99) and unnatural/external causes (including deaths from suicide and from accidents and assaults) (U509, V01-Y89) mortality and deaths not elsewhere classified (R00-R99). Cause-specific codes were further grouped as deaths from cancers (C00-D48), respiratory disorders (J00-J99), circulatory disorders (I00-I99), diabetes mellitus (E10-E14) and suicide (X60-X84, Y10-Y34). Using information on suicide and external causes, we created a further category for non-suicide external causes of death. We defined alcohol-related mortality using a standard set of ICD codes for fully and partially alcohol-attributable conditions to align with previous research and public health reporting. Although foetal alcohol syndrome is caused by maternal prenatal alcohol exposure rather than the decedent’s own drinking, we retained this code in the alcohol-related mortality category to capture all alcohol-attributable deaths:

E244: Alcohol-induced pseudo-Cushing's syndrome

F100-199: Mental and behavioural disorders due to use of alcohol

G312: Degeneration of nervous system due to alcohol

G621: Alcoholic polyneuropathy

G721: Alcoholic myopathy

I426: Alcoholic cardiomyopathy

K292: Alcoholic gastritis

K700-709: Alcoholic liver disease

K852: Alcohol-induced acute pancreatitis

K860: Alcohol-induced chronic pancreatitis

Q860: Foetal-induced alcohol syndrome (dysmorphic)

R780: Excess alcohol blood levels

X450-X459: Accidental poisoning by and exposure to alcohol

X650-659: Intentional self-poisoning by and exposure to alcohol

Y150-159: Poisoning by and exposure to alcohol

Language rules used to define the presence of a violent behaviour risk event

The following strings were used to search for text in risk event descriptions to classify a risk event involving violent behaviour, applied to the first year of available records:

Abus*

Aggress*

Agitat*

Arous*

Assault*

Demand*

Hit*

Restrain*

Shout*

Threat*

Threw/throw*

Violen*
